# Anticancer Profiling for Coumarins and Related *O*-Naphthoquinones from *Mansonia gagei* against Solid Tumor Cells In Vitro

**DOI:** 10.3390/molecules23051020

**Published:** 2018-04-26

**Authors:** Mohammed A. Baghdadi, Fahad A. Al-Abbasi, Ali M. El-Halawany, Ali H. Aseeri, Ahmed M. Al-Abd

**Affiliations:** 1 Department of Biochemistry, Faculty of Science, King Abdulaziz University, Jeddah 21589, Saudi Arabia; m-baghdadi@hotmail.com (M.A.B.); alabassif@hotmail.com (F.A.A.-A.); 2 Research Centre, King Faisal Specialist Hospital & Research Centre, Jeddah 21499, Saudi Arabia; 3 Pharmacognosy Department, Faculty of Pharmacy, Cairo University, Cairo 11562, Egypt; ahalawany2003@yahoo.com; 4 Stem Cell Research Unit, King Fahad Medical Research Center, King Abdulaziz University, Jeddah 21589, Saudi Arabia; 5 Ministry of Health, Jeddah 21484, Saudi Arabia; alhaseeri@moh.gov.sa; 6 Department of Pharmacology, Medical Division, National Research Centre, Cairo 12622, Egypt

**Keywords:** *O*-naphthoquinones, coumarins, mansorin-II, paclitaxel, *Mansonia gagei*, colorectal cancer, P-glycoprotein, cell cycle, J0101

## Abstract

Napthoquinones and coumarins are naturally occurring compounds with potential anticancer activity. In the current study, two *O*-naphthoquinons (mansonone-G and mansonone-N) and six coumarins (mansorin-A, mansorin-B, mansorin-C, mansorins-I, mansorin-II, and mansorin-III) were isolated from the heartwood of *Mansonia gagei* family Sterculariaceae. Isolated compounds were examined for their potential anticancer activity against breast (MCF-7), cervix (HeLa), colorectal (HCT-116) and liver (HepG2) cancer cells using Sulfarhodamine-B (SRB) assay. Mansorin-II and mansorin-III showed relatively promising cytotoxic profile in all cell lines under investigation with inhibitory concentrations (IC_50_s) in the range of 0.74 µM to 36 µM and 3.95 µM to 35.3 µM, respectively. In addition, mansorin-B, mansorin-C, mansorin-II and mansorin-III significantly increased cellular entrapment of the P-glycoprotein (P-gp) substrate, doxorubicin, in colorectal cancer cells expressing the P-gp pump. The inhibitory effect of the isolated compounds on P-gp pump was examined using human recombinant P-gp molecules attached to ATPase subunit. Mansorin-B and mansonone-G were found to inhibit the P-gp attached ATPase subunit. On the other hand, mansorin-C, mansorin-III and mansorin-II inhibited P-gp pump via dual action (P-gp related ATPase subunit inhibition and P-gp substrate binding site occupation). However, mansorin II was examined for its potential chemomodulatory effect to paclitaxel (PTX) against colorectal cancer cells (HCT-116 and CaCo-2). Mansorin-II significantly reduced the IC_50_ of PTX in HCT-116 cells from 27.9 ± 10.2 nM to 5.1 ± 1.9 nM (synergism with combination index of 0.44). Additionally, Mansorin-II significantly reduced the IC_50_ of PTX in CaCo-2 cells from 2.1 ± 0.8 µM to 0.13 ± 0.03 µM (synergism with combination index of 0.18). Furthermore, cell cycle analysis was studied after combination of mansorin-II with paclitaxel using DNA flow cytometry analysis. Synergism of mansorin-II and PTX was reflected in increasing apoptotic cell population in both HCT-116 and CaCo-2 cells compared to PTX treatment alone. Combination of mansorin-II with PTX in CaCo-2 cells significantly increased the cell population in G_2_/M phase (from 2.9 ± 0.3% to 7.7 ± 0.8%) with reciprocal decrease in G_0_/G1 cell fraction from 52.1 ± 1.1% to 45.5 ± 1.0%. Similarly in HCT-116 cells, mansorin-II with PTX significantly increased the cell population in G_2_/M phase (from 33.4 ± 2.8% to 37.6 ± 1.3%) with reciprocal decrease in the S-phase cell population from 22.8 ± 1.7% to 20.2 ± 0.8%. In conclusion, mansorin-II synergizes the anticancer effect of paclitaxel in colorectal cancer cells, which might be partially attributed to enhancing its cellular entrapment via inhibiting P-gp efflux pump.

## 1. Introduction

Naphthoquinones are a group of widely distributed phenolics of plant origin and are classified to ortho and para-naphthoquinones. Ortho-naphthoquinones are particularly known for their cytotoxic effects among other biological activities such as anti-bacterial, anti-fungal and antiparasitic and acetylcholine esterase inhibitory effects [1]. Mansonones are a group of sesquiterpene-derived ortho-naphthoquinones occurring in different genuses in the plant kingdom such as Hibiscus, Mansonia and Thespesia [2]. Mansonones are, biosynthetically, thought to be phytoalexins produced by the injury of bark of some plants such as American elm (*Ulmus minor*). Mansonones E and F were reported to possess a potent cytotoxic effect on HeLa, human malignant melanoma A357-S2, MCF-7 and human histocytic lymphoma U937 cell lines [3,4]. In addition, antileukemic and topoisomerase inhibitory effects were reported to mansonone E. However, other mansonones such as mansones A–D, G, I–S were not investigated for their possible cytotoxic effects on several cell lines despite their structural similarity to that of mansones E and F [5].

Coumarins are naturally occurring benzopyrones with wide biological activities reported such as anti-inflammatory, antibacterial, cytotoxic, antioxidant and anti HIV effects [6]. Several naturally occurring coumarins and their synthetic derivatives exhibited cytotoxic effects via a telomerase enzyme inhibitory effect, protein kinases inhibition and oncogene downregulation. Other suggested cytotoxic mechanisms of coumarins are induction of caspase-9-mediated apoptosis and antiproliferative effects due to cell cycle arrest in G_0_/G_1_-phase and G_2_/M-phase [7,8]. Finally, coumarins are expected to inhibit the efflux activity of p-glycoprotein (P-gp) and enhance the anticancer properties of several P-gp substrate chemotherapies [9]. 

*Mansonia gagei* is a tree belonging to the family Sterculiaceae and native to Thailand [10]. *M. gagei* heartwood was reported as a folk remedy for cardiac stimulation, anti-emetic, antidepressant and refreshing agent [11]. Several *O*-naphthoquinones named mansonones were isolated from this plant. In addition, a group of unique coumarins (Mansorin A–C and I–III), which are structurally related to mansonones, were also isolated from *M. gagei*. Mansonones and mansorins obtained from *M. gagei* revealed anti-estrogenic, larvicidal, antioxidant and antifungal activities [12,13,14,15,16]. Despite there are some reports about the cytotoxic effects of mansonones, there is nothing reported regarding the cytotoxicity of coumarins from this plant. Herein, we tested the potential cytotoxic effects of *O*-naphthoquinones and related coumarins from *M. gagei* against four different types of solid tumor cells. Among them, mansorin-II was further investigated for potential chemomodulatory effect to paclitaxel against colorectal cancer cells. 

## 2. Results and Discussion

### 2.1. Isolation and Structural Identification of O-Naphthoquinones and Coumarins 

**Figure 1:**
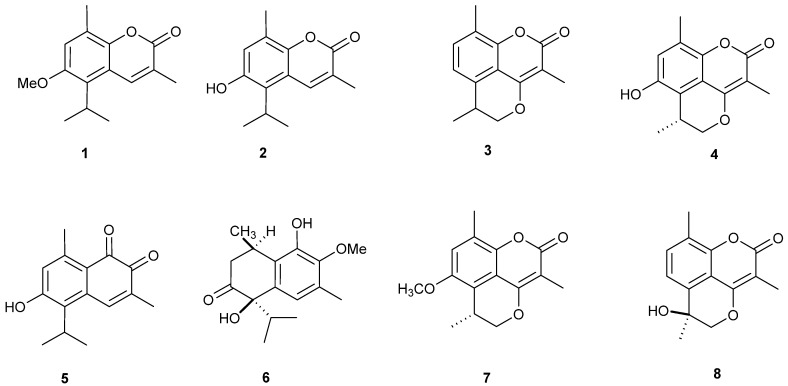
Compounds isolated from the heartwood of *M. gagie* Drumm.

The CHCl_3_-soluble fraction of *M. gagie* was subjected to several chromatographic processes using normal and reversed phase silica gel columns to obtain 7 compounds (**1**, **3**–**8**) (Figure 1). In addition, compound **2** was obtained from its closely related **1** by demethylation using hydroiodic acid (Figure 1). The compounds were identified as six coumarins; mansorin A (**1**), mansorin B (**2**), mansorin C (**3**) [13], mansorin I (**4**), mansorin II (**7**) and mansorin III (**8**) [16], and, in addition to two sesqueterpenoid naphthoquinones, mansonone G (**5**) [17], and mansonone N (**6**) [15]. Compounds were identified by comparing its ^1^H and ^13^C-NMR data with the reported literature (Supplementary Materials Figures S5–S12).

### 2.2. Cytotoxicity Assessment of Some O-Naphthoquinones and Coumarins 

SRB-U assay was used to assess the cytotoxicity of eight naturally naphthoquinone and related coumarin compounds against four different tumor cell lines over concentration range 0.01–100 μM. Tested compounds showed variable cytotoxicity against cell lines under investigation (HCT-116, HepG2, MCF-7 and HeLa cell lines) (Supplementary Materials Figures S1–S4). However, MCF-7 was relatively more resistant while HeLa was the most sensitive cell line under investigation (Table 1).

In HCT-116 colorectal cancer cells, mansorin-A, mansorin-I and mansorin-II showed the most potent cytotoxic profile with R-values less than 20% and IC_50_s of 11.2 μM, 11.1 μM and 19.3 μM, respectively. Mansonone-G, mansonone-N and mansorin-III possessed relatively weaker cytotoxic profile with IC_50_s higher than 50 μM. With respect to mansorin-B and mansorin-C, despite their apparent high potencies (IC_50_s of 5.7 μM and 8.6 μM, respectively), their R-values were higher than 20% (26.3% and 49.9%, respectively). It was found in our previous work that decreasing the R-value percent is an indicator of diminishing colorectal cancer cell resistance due to the overexpression of the P-glycoprotein efflux pump [18]. 

In HepG2 liver cancer cells, mansorin-B, mansorin I, mansonone-G and mansorin II showed relatively potent cytotoxic profile at an R-value less than 20% and IC_50_s ranging from 21.9 μM to 49.4 μM. Mansonone-N showed weak cytotoxicity with IC_50_ higher than 100 μM. However, the R-value of mansonone-N was relatively low (5.2%). On the other hand, mansorin-A, mansorin-C and mansorin III suffered from high cellular resistance in HepG2 cells (R-values were higher than 30%). Similar to colorectal cancer cells, liver cancer might express P-gp pump [19]. In addition, other underlying reasons for liver cancer cell resistance are reported such as tumor-associated stem cells [20], overexpression of oncogenes and/or downregulation of tumor suppressor genes [21]. 

With respect to MCF-7 breast cancer cells, mansorin I, mansonone-G and mansorin II showed considerable cytotoxicity with IC_50_s of 23.8 μM, 23.0 μM and 36.0 μM, respectively (R-values were less than 20%). Mansonone-N and mansorin I showed weak cytotoxicity with IC_50_ higher than 100 μM. Other compounds (mansorin-A, mansorin-B and mansorin-C) showed apparently low IC_50_s (2.1 μM, 5.0 μM and 3.1 μM, respectively) with very high resistance (R-values were higher than 70%).

The most sensitive cell line to *O*-naphthoquinones and related coumarins under investigation was HeLa cells; mansorin-A, mansorin-B, mansorin-C, mansorin II, mansorin-I, and mansonone-G killed HeLa cells with IC_50_s less than 50 μM and R-values less than 10%. Only mansonone-N did not show any cytotoxicity against HeLa cells with IC_50_ higher that 100 μM and mansorin-III suffered from resistant fraction higher than 30%. Structurally related coumarins are known to induce apoptosis and cell cycle arrest in cervical cancer cells such as HeLa cells [22]. 

Accordingly, mansorin-I and mansorin-II would be recommended for further investigations either as cytotoxic or cytotoxicity chemomodulating agent. It is worth mentioning that coumarins are known for their cytotoxic potential due to their non-covalent DNA binding ability [23]. 

### 2.3. The Influence of O-Naphthoquinones and Related Coumarins on the Cellular Pharmacokinetics within Tumor Cells

**Figure 2:**
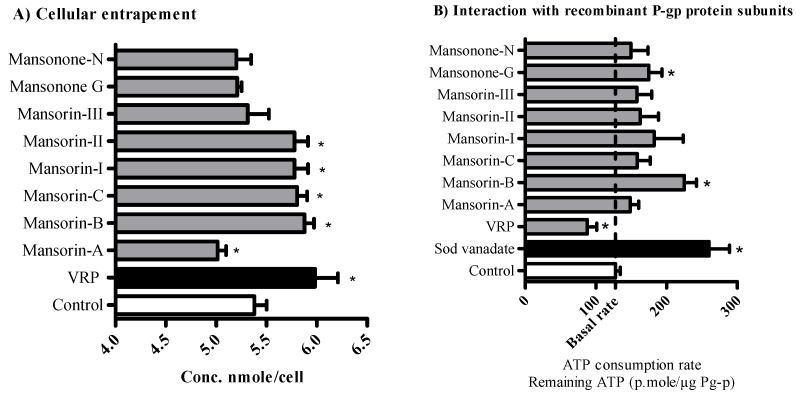
The effect of isolated compounds on the activity of P-glycoprotein efflux pump within HCT-116 cells (**A**) and in cell free isolated recombinant P-gp protein (**B**). Data is presented as mean ± SD; *n* = 3. (*): significantly different from CCl_4_ treated group.

Multidrug resistance in particular tumor types, such as solid tumors within the gastrointestinal tract, is highly attributed to impaired cellular pharmacokinetics and intracellular drug entrapment issues [24]. The ability of *O*-naphthoquinones and related coumarins to enhance the cellular entrapment of P-glycoprotein substrates was tested within HCT-116 colorectal cells. Mansorin-II, mansorin-B, mansorin-C and mansorin-I significantly increased cellular internalization of doxorubicin (P-gp probe) and significantly increased its intracellular concentration from 5.37 ± 0.17 nmole/cell to 5.78 ± 0.19 nmole/cell, 5.55 ± 0.13 nmole/cell, 5.8 ± 0.14 nmole/cell and 5.78 ± 0.19 nmole/cell, respectively. On the other hand, mansorin-A decreased the intracellular concentration of doxorubicin to 5.01 ± 0.12 nmole/cell (Figure 2A). HCT-116 and other colorectal cancer cells are known to express P-gp efflux protein and participate considerably in their resistance to chemotherapies [18,25]. 

Further investigation for the sub-molecular interaction between the isolated compounds and P-gp subunits was undertaken using human recombinant P-gp membrane bound protein linked to ATPase enzyme subunits. P-gp binding site inhibitors such as verapamil (VRP) are supposed to increase ATPase activity due to conformational changes and results in more ATP consumption (68.7% less remaining ATP concentration compared to basal ATP consumption). On the other hand, direct ATPase enzyme subunit inhibitors such as sodium vanadate would decrease ATP consumption (203.7% more remaining ATP concentration compared to basal consumption condition). Only mansorin-B and mansonone-G showed pure ATPase inhibitory effects with 155.6% and 137.0% more remaining ATP concentration, respectively (Figure 2B). Other naphthoquinones and coumarins did not induce any significant change for ATP consumption rate. This might be attributed to lack of interaction with either subunit of P-gp molecules or attributed to dual interaction with both subunits. However, mansorin-II, mansorin-C and mansorin-I significantly increased cellular entrapment of P-gp probe. Accordingly, it is suggested that mansorin-II, mansorin-C and mansorin-I interact with both subunits of P-gp molecules. On the other hand, mansorin-A, mansonone-N and mansorin-III did not exert any significant interaction with either subunits of P-gp molecules. Our previous work on synthetic compounds based on curcumin scaffold showed a distinct ATPase inhibitory effect and/or binding site inhibition effect. This, in turn, increased the efficacy and decreased the resistance to paclitaxel within colorectal cancer cells [18]. Many other P-gp inhibitors of synthetic origin were suggested as potential chemomodulators of several anticancer drugs [26,27,28,29]. Herein, a similar chemomodulatory effect is expected when combining compounds such as mansorin-II with P-gp substrate (such as paclitaxel) [30]. However, several compounds of natural origin are known for their P-gp inhibitory potential and hence chemomodulatory capacity due to improving the cellular pharmacokinetics of anticancer agents [9,31,32].

### 2.4. Chemomodulatory Effect of Mansorin-II to Paclitaxel (PTX) against Colorectal Cancer Cells

**Figure 3:**
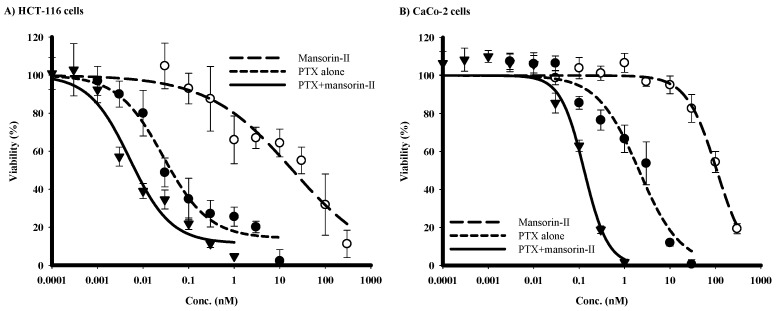
The effect of mansorin-II on the cytotoxicity of PTX in HCT-116 (**A**) and CaCo-2 (**B**) cell lines. Cells were exposed to serial dilution of PTX (●), mansorin-II (○) or their combination (▼) for 72 h. Cell viability was determined using SRB assay.

P-gp efflux activity is a considerable determinant for paclitaxel intracellular pharmacokinetics and hence cell killing effect [30,33]. Attributed to its considerable cytotoxic profile and its P-gp interaction properties, mansorin-II would be a good candidate to improve the activity of P-gp substrate drugs (such as paclitaxel) within P-gp expressing tumor cell types (such as colorectal cancer). Cytotoxicity of mansorin-II was much weaker in CaCo-2 cells compared to HCT-116 cells with IC_50_s of 107.9 ± 6.4 µM and 19.3 ± 3.7 µM, respectively. However, mansorin-II synergistically improved the cytotoxic profile of PTX against both colorectal cancer cell lines. Mansorin-II significantly decreased the IC_50_s of PTX form 27.9 ± 10.2 nM and 2.1 ± 0.8 µM to 5.1 ± 1.9 nM and 0.13 ± 0.03 µM in HCT-116 and CaCo-2 cells, respectively (Figure 3A,B). The combination indices for equitoxic combination of mansorin-II and PTX within HCT-116 and CaCo-2 cells were 0.44 and 0.18, respectively. In addition, mansorin-II decreased the resistant fraction to PTX within HCT-116 and CaCo-2 cells from 14.2 ± 5.3% and 3.1 ± 0.37% to 11.6 ± 6.3% and 1.8 ± 0.25%, respectively (Table 2). As shown in our previous work, inhibiting P-gp ATPase subunit would decrease tumor cell resistance, while binding site inhibition increased anticancer potency (decreased IC_50_) [18].

### 2.5. Cell Cycle Distribution Analysis of Colorectal Cancer Cells

**Figure 4:**
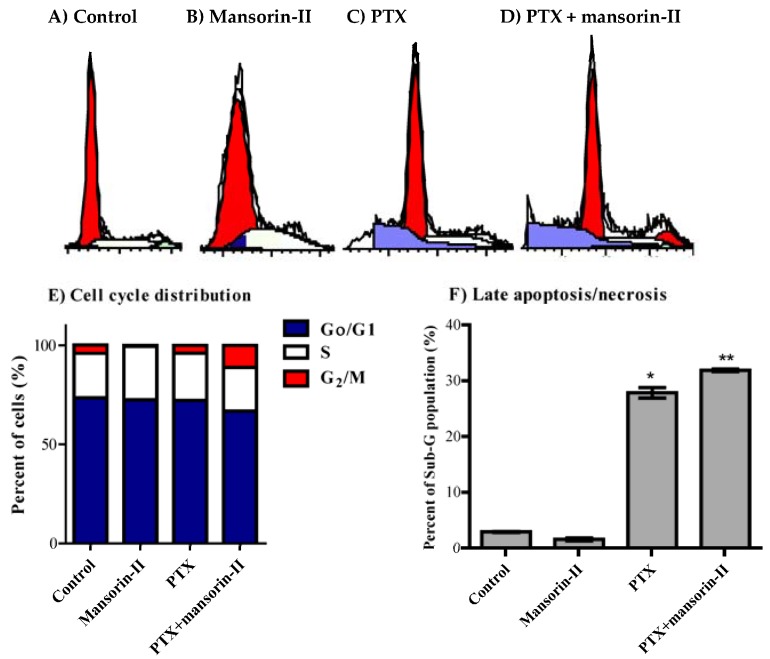
Effect of mansorin II on the cell cycle distribution of CaCo-2 cells. The cells were exposed to mansorin II (**B**), PTX (**C**), or combination of mansorin II and PTX (**D**) for 24 h and compared to control cells (**A**). Cell cycle distribution was determined using DNA cytometry analysis and different cell phases were plotted (**E**) as percentage of total events. Sub-G cell population was taken as representative of late apoptosis/necrosis and was plotted as percent of total events (**F**). Data is presented as mean ± SD; *n* = 3. (*): significantly different from control group; (**): significantly different from PTX group.

Cell cycle distribution using DNA flow cytometry was used to investigate the nature of interaction between PTX and mansorin-II. Cell cycle analysis can figure out or dissect the antiproliferative effect of PTX, mansorin-II or their combination in terms of cell cycle phase specificity (G_0_/G_1_, S and G_2_/M-phases) [34]. In addition, it gives considerable clues for potential cell killing effects by assessing cell population in the pre-G phase [35]. In CaCo-2 cells, mansorin II significantly increased cell population is S-phase from 22.4 ± 1.4% to 29.9 ± 0.6% with reciprocal decrease inG_2_/M cell population (Figure 4A,B,E). Suboptimal exposure (24 h) to PTX alone did not exert any significant change in the cell cycle distribution pattern of CaCo-2 cells (Figure 4A,C,E). Combination of PTX with mansorin-II induced significant cell cycle arrest at G_2_/M phase (11.2 ± 0.7%) compared to control or PTX treatment alone (4.0 ± 0.3% and 4.2 ± 0.4%, respectively). The induced G_2_/M cell phase arrest was accompanied by reciprocal decrease in the non-proliferating cell population (G_0_/G_1_-phase) from 73.4 ± 1.1% to 66.8 ± 0.8% (Figure 4A,C–E). Finally, exposure of CaCo-2 cells to PTX for 24 h resulted in significant cell death observed by elevated pre-G cell population form 2.9 ± 1.1% to 27.8 ± 0.9%. Combination of PTX with mansorin-II further increased pre-G cell population to 31.9 ± 0.3% (Figure 4F).

**Figure 5:**
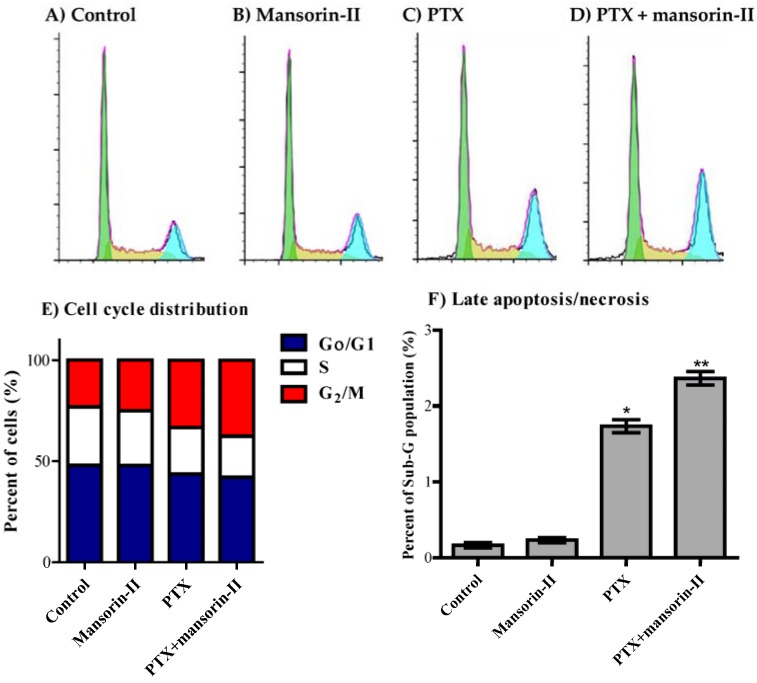
Effect of mansorin II on the cell cycle distribution of HCT-116 cells. The cells were exposed to mansorin II (**B**), PTX (**C**), or combination of mansorin II and PTX (**D**) for 24 h and compared to control cells (**A**). Cell cycle distribution was determined using DNA cytometry analysis and different cell phases were plotted (**E**) as percentage of total events. Sub-G cell population was taken as representative of late apoptosis/necrosis and was plotted as percent of total events (**F**). Data is presented as mean ± SD; *n* = 3. (*): significantly different from control group; (**): significantly different from PTX group.

With respect to HCT-116, mansorin-II significantly decreased cell population is S-phase from 29.0 ± 0.4% to 27.3 ± 0.7% with reciprocal apparent increase in G_2_/M cell population from 23.1 ± 0.5% to 25.0 ± 0.9% (Figure 5A,B,E). PTX alone significantly induced cell cycle arrest at G_2_/M-phase (33.4 ± 1.6%) with reciprocal decrease in both S-phase and G_0_/G1-phase from 29.0 ± 0.4% to 22.8 ± 1.0% and from 47.8 ± 0.8% to 43.8 ± 0.9%, respectively (Figure 5A,C,E). Combination of mansorin II with PTX further increased cell accumulation in G_2_/M phase up to 37.6 ± 0.8% with further reciprocal decrease in S-phase to 20.2 ± 0.4% (Figure 5A,D,E). Finally, exposure of HCT-116 cells to PTX for 24 h resulted in significant cell death observed by elevated pre-G cell population form 0.2 ± 0.01% to 1.7 ± 0.1%. Combination of PTX with mansorin II further increased pre-G cell population to 2.4 ± 0.1% (Figure 5F).

Coumarins were found to induce cell cycle arrest and cell killing effect in tumor cells [22]. Herein, mansorin-II potentiated the PTX-dependent cell cycle arrest and cell killing effects. Despite some reports for the potential anticancer properties of natural and synthetic mansorins [36,37,38], to the best of our knowledge, this is the first report for anticancer chemotherapeutic and chemomodulatory effect of mansorin-II.

## 3. Materials and Methods 

### 3.1. Drugs and Chemicals

Verapamil (VRP), trypan blue and Sulforhodamine-B (SRB) were purchased from Sigma Chemical Co. (St. Louis, MO, USA). Phosphate buffer saline (PBS) was purchased from Becton Dickinson (Fullerton, CA, USA). Penicillin/streptomycin and trypsin, RPMI-1640 media, DMEM media, fetal bovine serum (FBS), and other cell culture materials were purchased from Gibco (Grand Island, NY, USA). Other reagents were of the highest analytical grade.

### 3.2. General Experimental Procedures

TLC analysis was performed on pre-coated TLC plates with silica gel 60 F_254_ (Merck, Darmstadt, Germany). Column chromatographic separations were performed on silica gel 60 (70–230 mesh, Merck, Darmstadt, Germany). ^1^H and ^13^C-NMR spectra were recorded on a Bruker DRX-850 MHz Ultrashield spectrometer (Bruker BioSpin, Billerica, MA, USA) using CDCl_3_ as solvent, with TMS as the internal reference. Medium pressure liquid chromatography (MPLC) was performed on LiChroprep RP-18 and LiChroprep Si 60 (size A and B, Merck Co., Kenilworth, NJ, USA).

### 3.3. Plant Material

The heartwood of *Mansonia gagei* Drumm was bought from the herbal drugstore “Cho Krom Pur,” Bangkok, Thailand, and was identified by Dr. Katsuko Komatsu (Institute of Natural Medicine, University of Toyama, Toyama, Japan). A voucher specimen has been kept in the herbarium of the Institute of Natural Medicine, University of Toyama, Japan.

### 3.4. Extraction and Isolation of Compounds from A. melegueta

The mansonones and mansorins were isolated as previously described [16]. The dried powdered heartwood of *M. gagei* (3.5 kg) was extracted with methanol on cold. The methanol extract (250 g) was suspended in water (500 mL) and partitioned with chloroform (1L × 3) and the pooled chloroform fractions were evaporated under vacuum. The combined chloroform fraction (100 g) was fractionated on a silica gel column (70 cm × 8 cm) eluted with hexane-acetone (5% until 80% *v*/*v*) to yield eight fractions. Fraction 1 (7 g) was applied to a silica gel column (40 cm × 4 cm) eluted with hexane-ethyl acetate (9.5:0.5 *v*/*v*) to obtain compound **1** (4 g). The remaining of fraction 1 was re-chromatographed using an MPLC silica gel 60 column (size A) (hexane-ethyl acetate, 9.5:0.5 *v*/*v*) to afford compounds **3** (15 mg), **6** (30 mg) and **7** (16 mg). Fraction 3 (5.2 g) was applied to a silica gel column (40 cm × 4 cm) and eluted with hexane-ethyl acetate (9.5:0.5~9:1 *v*/*v*), then the eluate was pooled into three main sub-fractions. Fraction 3-2 was purified on a silica gel column (20 cm × 2.5 cm) eluted with hexane-ethyl acetate (9:1 *v*/*v*) to obtain compound **5** (2 g). Fraction 3-3 was purified on an MPLC RP-18 column (size B) eluted with MeOH-H_2_O (7:3 *v*/*v*) to get compound **4** (7 mg) and **8** (15 mg). 

### 3.5. Preparation of Compound ***2***

Compound **1** (116 mg, 0.5 mmol) was dissolved in acetic anhydride (5 mL). Hydroiodic acid (HI) (d = 1.7, 2 mL) was added to the previous solution with occasional shaking. The mixture was refluxed at 150 °C for 2 h in an oil bath. The resultant product was added to a saturated solution of sodium thiosulfate and extracted with ethyl acetate (10 mL × 3). The pooled ethyl acetate extracts were applied to a silica gel column using n-hexane-EtOAc (9.5:0.5 *v*/*v*) to obtain **2** as a yellowish white powder (91.5 mg) (74%) [39].

### 3.6. Cell Culture

Five different human solid tumor cell lines were used; colorectal cancer cell lines (HCT-116 and CaCo-2), cervical cancer cell line (HeLa), hepatocellular canrcinoma cell line (HepG2), and breast adenocarcinoma cell line (MCF-7). All cell lines were obtained from VACSERA, Giza, Egypt. Cell lines were maintained in RPMI-1640 or DMEM media containing 100 U/mL penicillin; 100 µg/mL streptomycin, and supplemented with 10% heat-inactivated fetal bovine serum (FBS). Cells were propagated in a humidified cell culture incubator with 5% (*v*/*v*) CO_2_ at 37 °C. 

### 3.7. Cytotoxicity Assessment

The cytotoxicity of the isolated compounds was tested against HCT-116, HeLa, HepG2, and MCF-7 cells by SRB assay as previously described [40]. Briefly, exponentially growing cells were collected using 0.25% Trypsin-EDTA and seeded in 96-well plates at 1000–2000 cells/well. Cells were treated with the isolated compounds for 72 h and subsequently fixed with trichloroacetic acid, TCA (10%) for 1 h at 4 °C. After several washings with water, cells were exposed to 0.4% SRB solution for 10 min at room temperature in a dark place and subsequently washed with 1% glacial acetic acid. After the plates dried overnight, Tris-HCl was used to dissolve the SRB stained cells. Color intensity was measured at 540 nm with SpectraMax® ELISA microplate reader (Molecular Devices LLC, San Jose, CA, USA).

### 3.8. Data Analysis 

The dose-response curves were analyzed as previously described [41] using E_max_ model (Equation (1)):

(1) $$\%\;\mathrm{Cell}\;\mathrm{viability}=\left(100-R\right)\times \left(1-\frac{{\left[D\right]}^{m}}{K_{d}{}^{m}+{\left[D\right]}^{m}}\right)+R,$$

where [R] is the residual unaffected fraction (the resistance fraction), [D] is the drug concentration used, [K_d_] or IC_50_ is the drug concentration that produces a 50% reduction of the maximum inhibition rate and [m] is a Hill-type coefficient. Absolute IC_50_ is defined as the drug concentration required to reduce absorbance by 50% of that of the control (i.e., K_d_ = absolute IC_50_ when R = 0 and E_max_ = 100-R).

### 3.9. The Influence of Mansorin-II and Other O-Naphthoquinones/Coumarins on the Cellular Pharmacokinetics

To assess the effect of mansorin-II and other naphthoquinones/coumarins on cellular pharmacokinetics in colorectal cancer cells, their effect on the efflux pumping activity of P-gp was evaluated. Herein, doxorubicin (DOX) was used as P-gp fluorescent substrate. Intracellular DOX concentration was determined with and without co-exposure with mansorin-II and other naphthoquinones/coumarins and compared to VRP as a standard P-gp inhibitor (positive control). Briefly, exponentially proliferating cells were plated in 6-well plates at plating density of 10^5^ cells/well. Cells were exposed to equimolar concentration of DOX (10 µM) and test compounds or VRP for 24 h at 37 °C and, subsequently, extracellular DOX-containing media was washed three times in ice cold PBS. Intracellular DOX was extracted after cell lysis by sonication with saturated aqueous solution of ZnSO_4_ (100 µL), acetonitril (500 µL) and acetone (250 µL) for 20 min at 37 °C. After centrifugation, clear supernatant solution was collected and DOX concentration was measured spectroflourometrically at λ_ex/em_ of 482/550 nm. DOX concentration was normalized based on cell number [42].

### 3.10. Determining Sub-Molecular Interaction Characteristics between P-gp Protein and O-Naphthoquinones/Coumarins

P-gp inhibitors block its efflux pumping activity via either covalent binding or inhibiting P-gp ATPase activity. Human recombinant membrane bound P-gp protein attached with ATPase subunit (Pgp-Glo™ Assay Systems, Promega Corporation, Madison, WI, USA) was used as previously described to determine the mechanism of P-gp inhibition via determining ATP consumption rate [18,25]. Briefly, test compounds (10 µM) were incubated with Pgp-Glo™ assay systems according to manufacturer’s protocol. Rate of ATP consumption was calculated by measuring the luminescent signal of the unmetabolized ATP via a firefly luciferase system. Compounds whose covalents bind to P-gp substrate binding sites are supposed to stimulate ATPase subunits and increase ATP consumption, while ATPase inhibitor compounds would decrease ATPase subunit activity and decrease the ATP consumption rate. Verapamil and sodium vanadate were used as positive controls (binding site blocker and ATPase inhibitors, respectively). ATP consumption was expressed as remaining ATP concentration and normalized per P-gp protein concentration (p.mole ATP/µg P-gp protein).

### 3.11. Chemomodulatory Effect of Mansorin-II to Paclitaxel within Colorectal Cancer Cells

The chemomodulatory effect of mansorin-II to paclitaxel (PTX) within colorectal cancer cells was determined using combination analysis between PTX and mansorin II as previously described [43]. Briefly, exponentially growing HCT-116 and CaCo-2 cells were seeded in 96-well plates (2000 cells/well) and exposed to equitoxic concentrations of PTX and mansorin-II for 72 h. Cells were subsequently subjected to SRB assay as described in the previous section. Combination index (CI-value) was calculated and used to define the nature of drug interaction (synergism if CI-value < 0.8 as; antagonism if CI-value > 1.2; and additive if CI-value ranges from 0.8–1.2). 

CI-value was calculated from the formula:

(2) $$CI-value=\frac{IC_{50}ofdrug(x)combination}{IC_{50}ofdrug(x)alone}+\frac{IC_{50}ofdrug(y)combination}{IC_{50}ofdrug(y)alone}.$$

### 3.12. Analysis of Cell Cycle Distribution

To assess the effect of the paclitaxel, mansorin-II and their combination on cell cycle distribution, CaCo-2 and HCT-116 cells were treated with the pre-determined IC_50_s of both agents for 24 h. After treatment, cells were collected by trypsinization, washed twice with ice-cold PBS and re-suspended in 0.5 mL of PBS. Two milliliters of 70% ice-cold ethanol were added gently while vortexing. Cells were kept in ethanol solution at 4 °C for 1 h for fixation. Upon analysis, fixed cells were washed and re-suspended in 1 mL of PBS containing 50 μg/mL RNAase A and 10 μg/mL propidium iodide (PI). After 20 min incubation in a dark place at room temperature, CaCo-2 cells were analyzed for DNA contents by FACS-VantageTM (Becton Dickinson Immunocytometry Systems, San Jose, CA, USA). For each sample, 10,000 events were acquired. Cell cycle distribution was calculated using CELLQuest software (Becton Dickinson Immunocytometry Systems) [44]. HCT-116 cells were injected through a ACEA Novocyte™ flow cytometer (ACEA Biosciences Inc., San Diego, CA, USA) and analyzed for DNA content using an FL2 signal detector (λ_ex/em_ 535/617 nm). For each sample, 12,000 events were acquired and quantified by ACEA NovoExpress™ software (ACEA Biosciences Inc., San Diego, CA, USA) [45].

### 3.13. Statistical Analysis

Data are presented as mean ± SEM using GraphPad prism™ software (version 5.00, GraphPad software Inc., La Jolla, CA, USA) for Windows. Analysis of variance (ANOVA) with a Newman–Keuls post hoc test was used for testing the significance using SPSS^®^ for Windows, version 17.0.0. (SPSS Inc., Chicago, IL, USA) *p* < 0.05 was taken as a cut-off value for significance.

## 4. Conclusions

Mansorin-II (naturally occurring coumarin) synergizes the anticancer effect of paclitaxel. This synergism might be partly attributed to interfering with the efflux activity of the P-gp pump and/or interfering with cell cycle progression. Further mechanistic studies for the proposed intracellular targets of mansorin-II and related compounds are strongly recommended.

## Figures and Tables

**Table 1:** Cytotoxicity parameters of some naturally occurring coumarins and *O*-naphthoquinones against different solid tumor cell lines.

Compound	HCT-116		HepG2		MCF-7		HeLa	
	IC_50_ (µM)	R-Fraction (%)	IC_50_ (µM)	R-Fraction (%)	IC_50_ (µM)	R-Fraction (%)	IC_50_ (µM)	R-Fraction (%)
Mansorin-A (**1**)	11.2	0.0	3.9	46.5	2.1	90.5	12.3	1.5
Mansorin-B (**2**)	5.7	26.3	21.9	0.0	5.0	78.9	38.7	1.3
Mansorin-C (**3**)	8.6	49.9	12.1	31.3	3.1	77.2	1.0	3.5
Mansorin-I (**4**)	11.1	0.0	35.3	0.0	23.8	0.0	3.95	0.0
Mansorin-II (**7**)	19.3	0.36	26.8	0.0	36.0	0.0	0.74	5.7
Mansorin III (**8**)	>100	0.0	7.2	69.6	>100	0.0	5.2	39.8
Mansonone-G (**5**)	63.4	0.5	49.4	1.1	23.0	5.1	18.8	1.8
Mansonone-N (**6**)	>100	0.0	>100	5.2	>100	97.2	>100	0.0

**Table 2:** Effect of mansorin-II on the cytotoxicity parameters of PTX in colorectal cancer cell lines.

	HCT-116		CaCo-2	
	IC_50_	R-Value (%)	IC_50_	R-Value (%)
**PTX**	27.9 ± 10.2 nM	14.2 ± 5.3	2.1 ± 0.8 µM	3.1 ± 0.37
**Mansorin-II**	19.3 ± 3.7 µM	0.36 ± 0.007	107.9 ± 6.4 µM	0.34 ± 0.004
**PTX with mansorin-II**	5.1 ± 1.9 nM	11.6 ± 6.3	0.13 ± 0.03 µM	1.8 ± 0.25
**CI-index/CI-value**	**Synergism/0.44**		**Synergism/0.18**	

## Supplementary Materials

The following are available online. Figure S1. Dose response curves of different naturally occurring *O*-naphthoquinones and related coumarins against HCT-116 cells, Figure S2. Dose response curves of different naturally occurring *O*-naphthoquinones and related coumarins against HepG2 cells, Figure S3. Dose response curves of different naturally occurring *O*-naphthoquinones and related coumarins against MCF-7 cells, Figure S4. Dose response curves of different naturally occurring *O*-naphthoquinones and related coumarins against HeLa cells, Figure S5. ^1^H- and ^13^C-NMR charts of compound **1**, Figure S6. ^1^H- and ^13^C-NMR charts of compound **2**, Figure S7. ^1^H and ^13^C NMR charts of compound **3**, Figure S8. ^1^H- and ^13^C-NMR charts of compound **4**, Figure S9. ^1^H- and ^13^C-NMR charts of compound **5**, Figure S10. ^1^H and ^13^C-NMR charts of compound **6**, Figure S11. ^1^H- and ^13^C-NMR charts of compound **7**, Figure S12. ^1^H- and ^13^C-NMR charts of compound **8**.

## Abbreviations

The following abbreviations are used in this manuscript:

CI-value	Combination Index
DOX	Doxorubicin
FBS	Fetal bovine serum
P-gp	P-glycoprotein
PBS	Phosphate buffer saline
PTX	Paclitaxel
SRB	Sulforhodamine-B
VRP	Verapamil
