# Supplementary material for: Anticancer Profiling for Coumarins and Related O-Naphthoquinones from Mansonia gagei against Solid Tumor Cells In Vitro
Source: Molecules. 2018 Apr 26;23(5):1020. doi: 10.3390/molecules23051020 (PMC6102575; doi:10.3390/molecules23051020)
Supplement: Supplementary file 1 [file molecules-23-01020-s001.pdf]

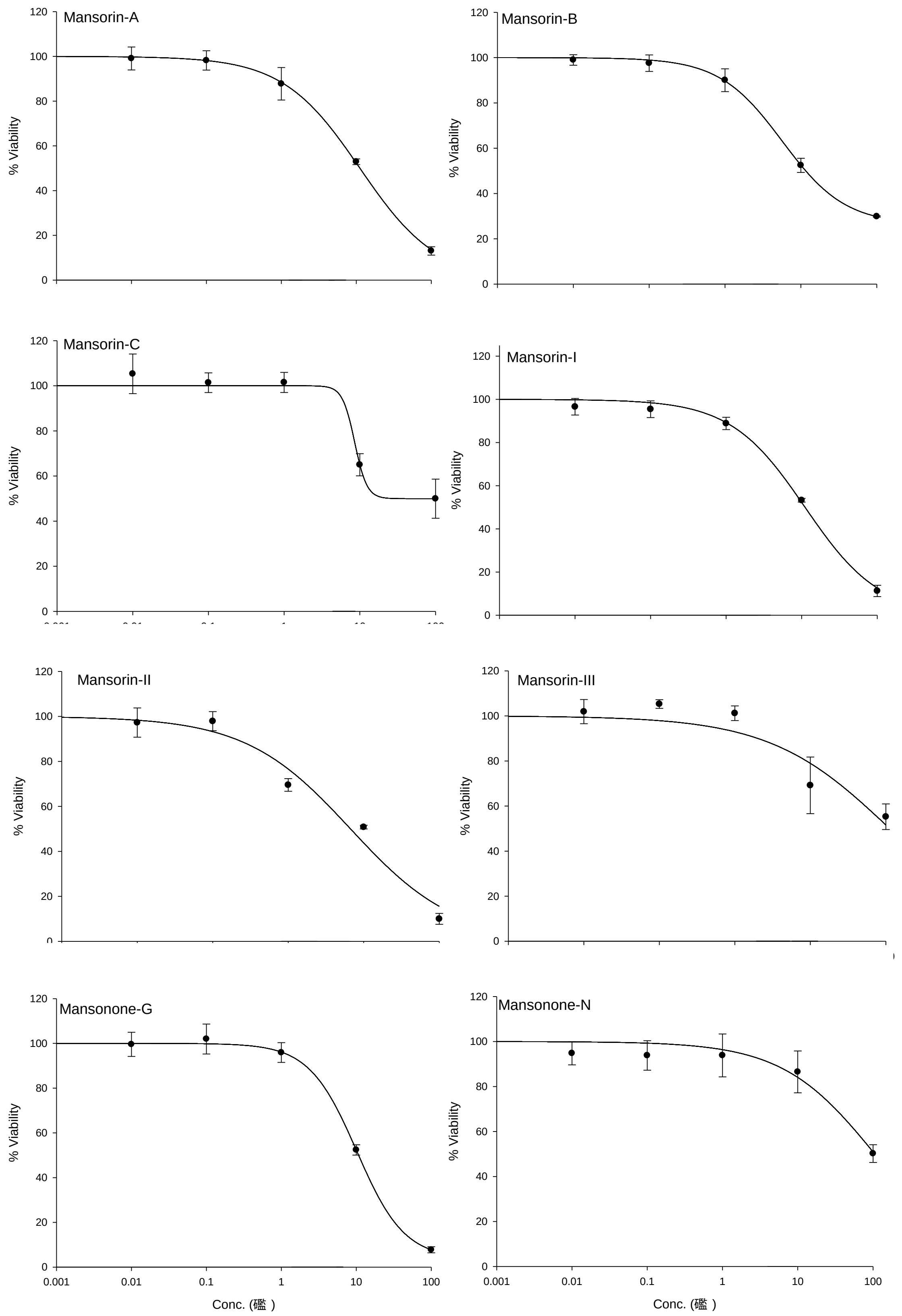

Supp. Fig. 1. Dose response curves of different naturally occurring coumarins and *O*-naphthoquinones against HCT-116 cells.

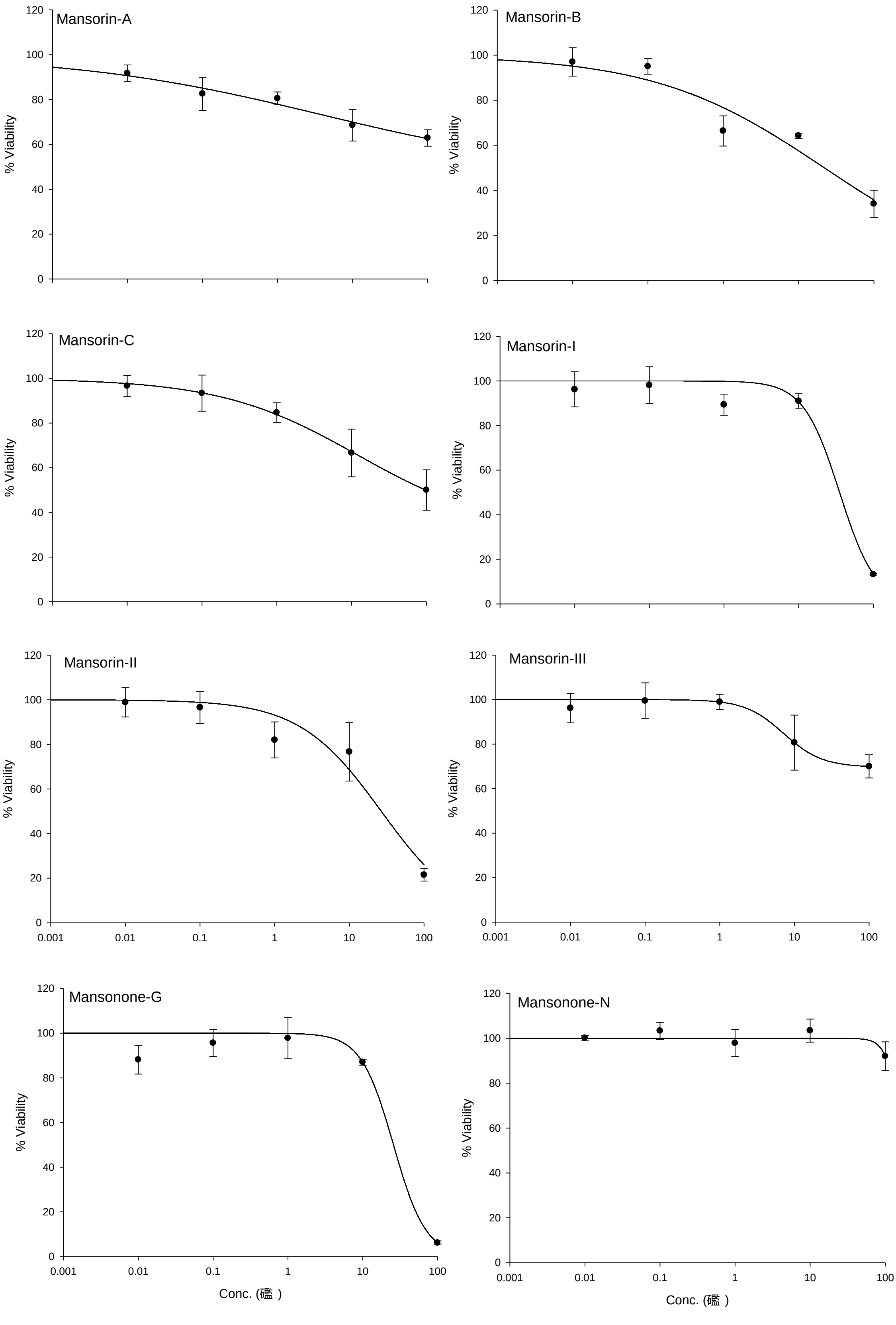

Supp. Fig. 2. Dose response curves of different naturally occurring coumarins and *O*-naphthoquinones against HepG2 cells.

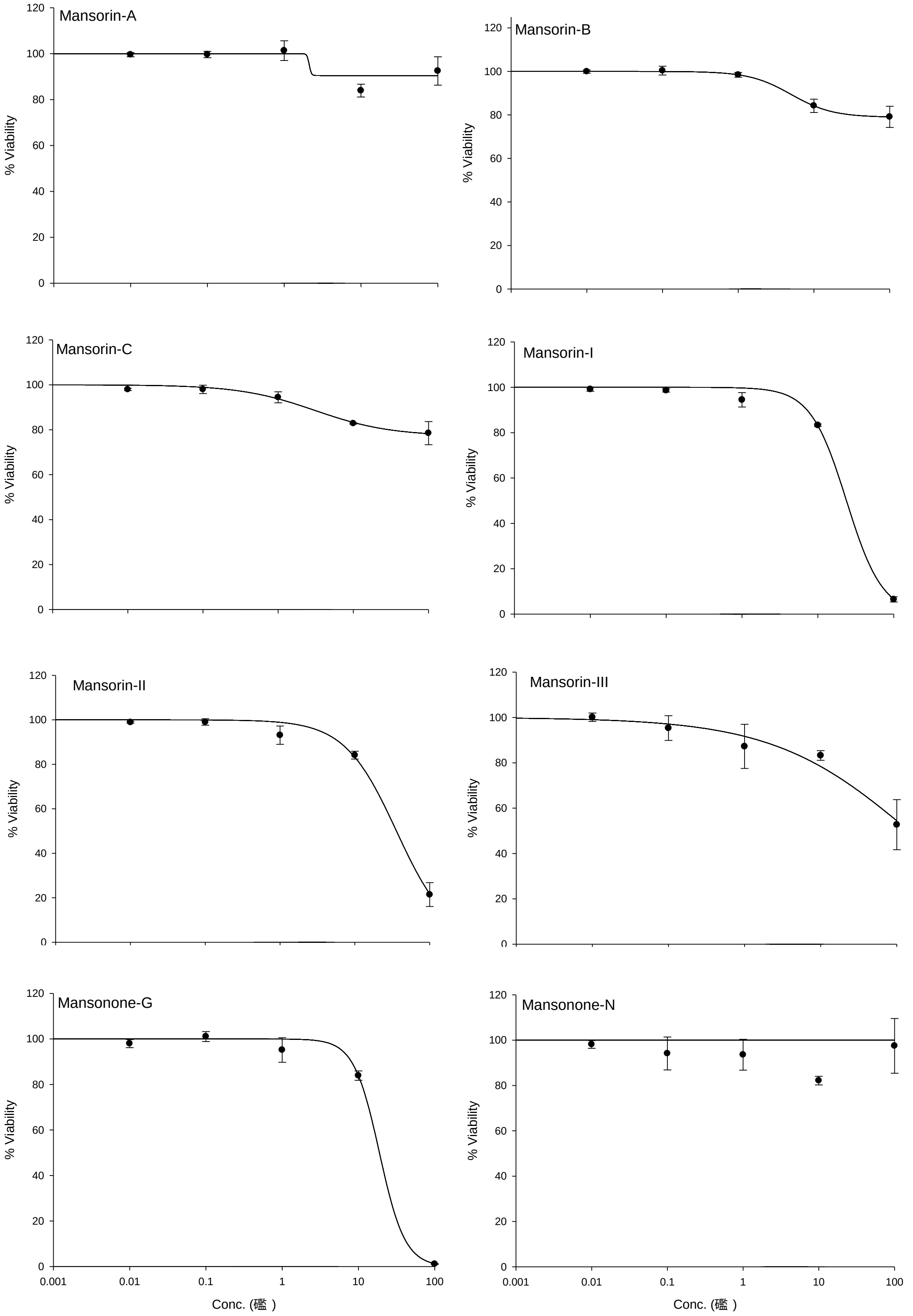

Supp. Fig. 3. Dose response curves of different naturally occurring coumarins and *O*-naphthoquinones against MCF-7 cells.

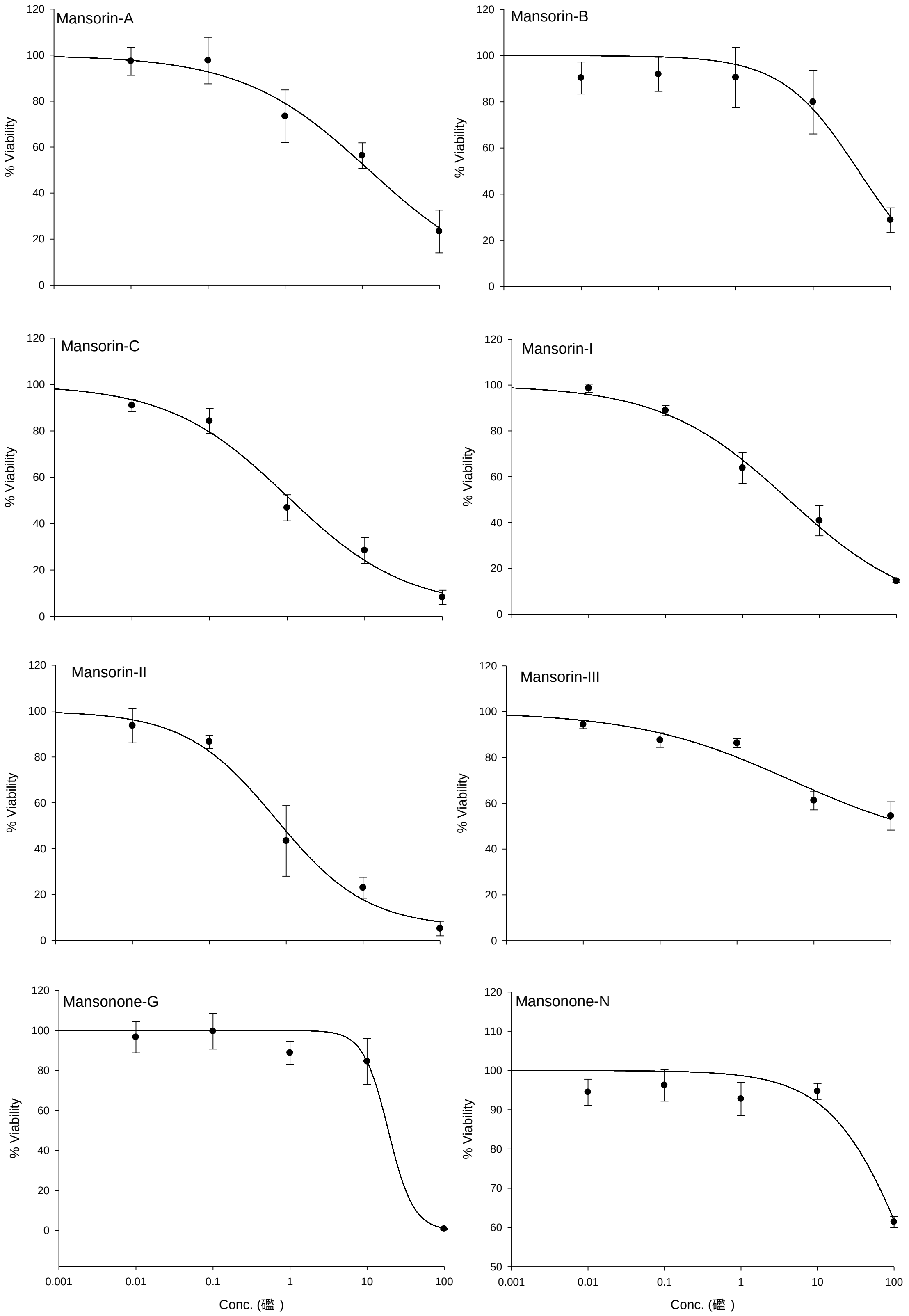

Supp. Fig. 4. Dose response curves of different naturally occurring coumarins and *O*-naphthoquinones against HeLa cells.

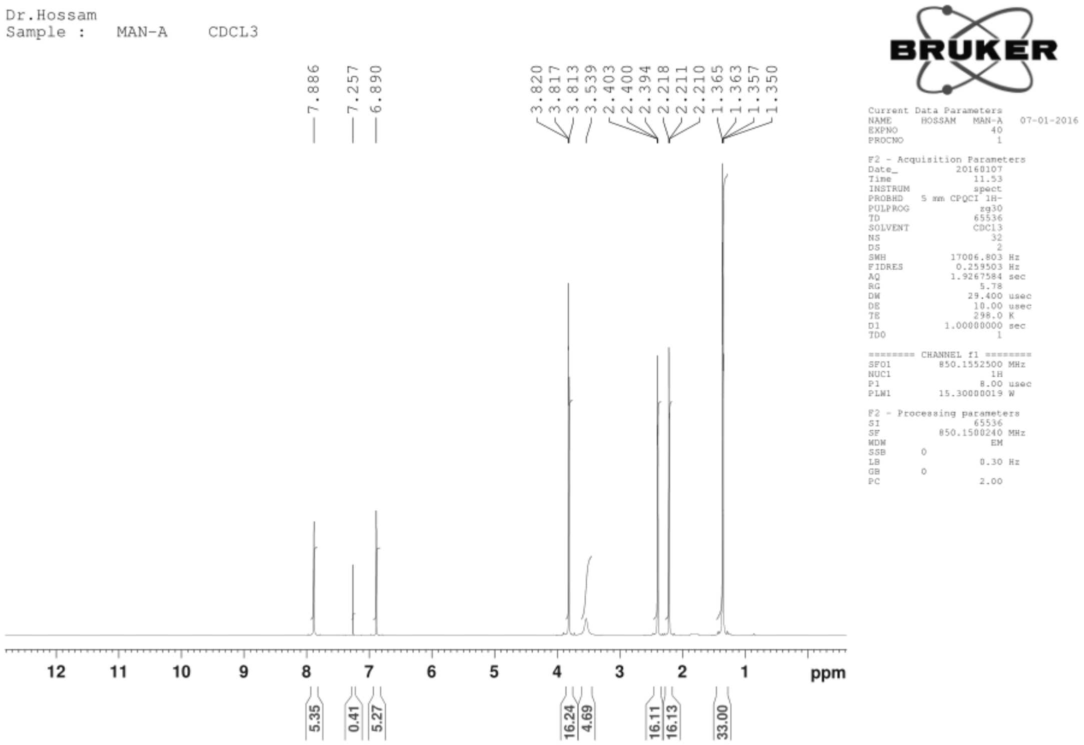

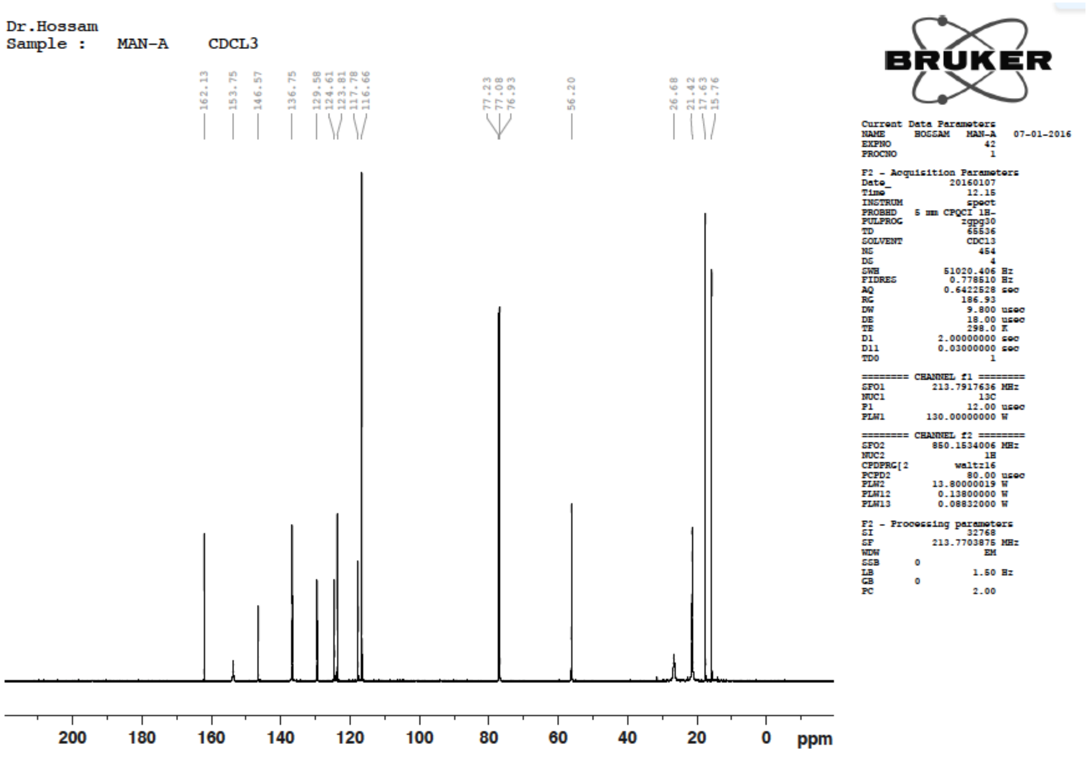

Supp. Fig 5. ^{1}H and [13] C NMR charts of compound 1

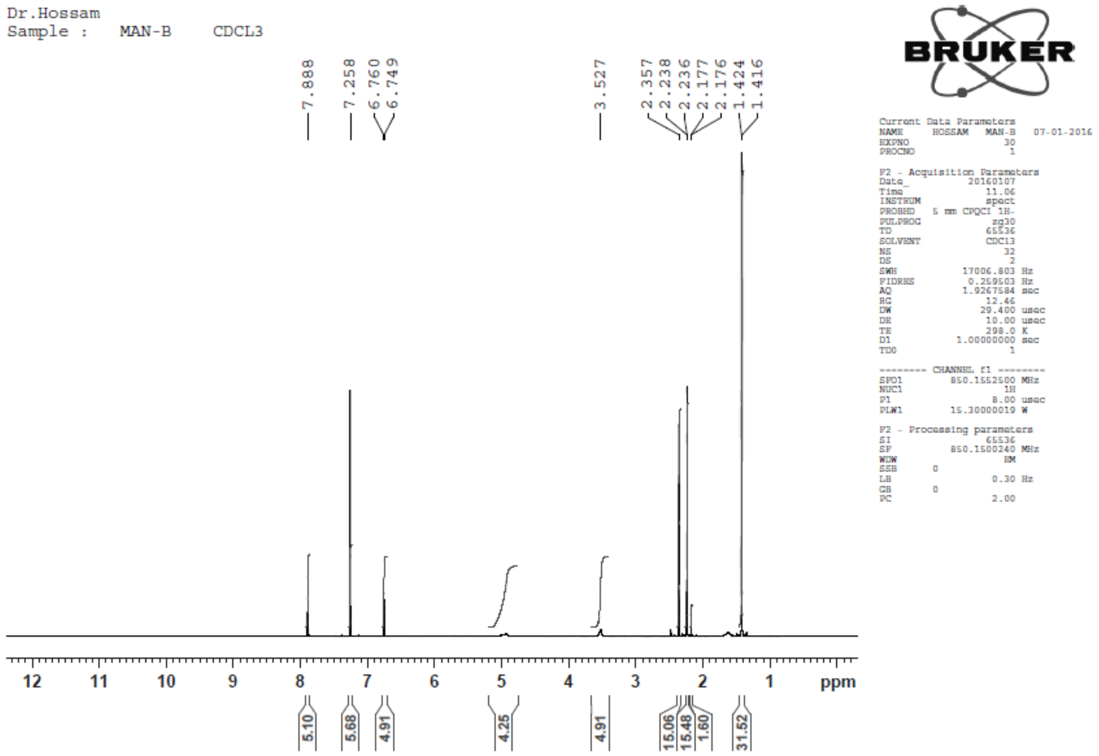

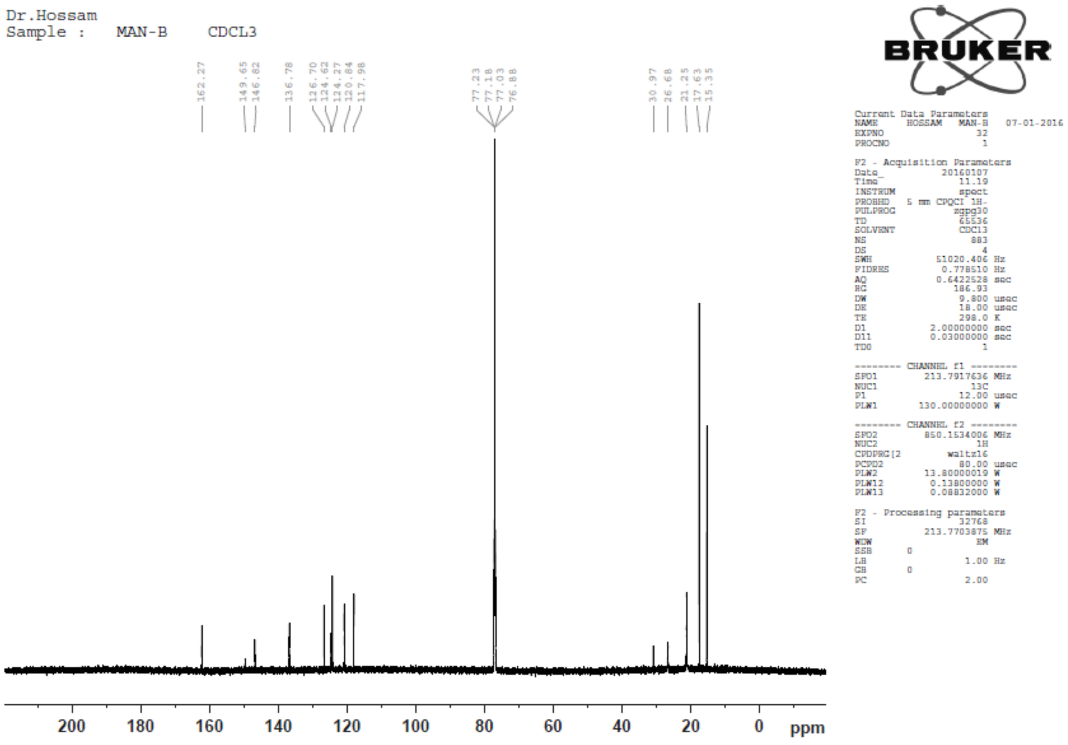

Supp. Fig 6. ^{1}H and [13] C NMR charts of compound 2

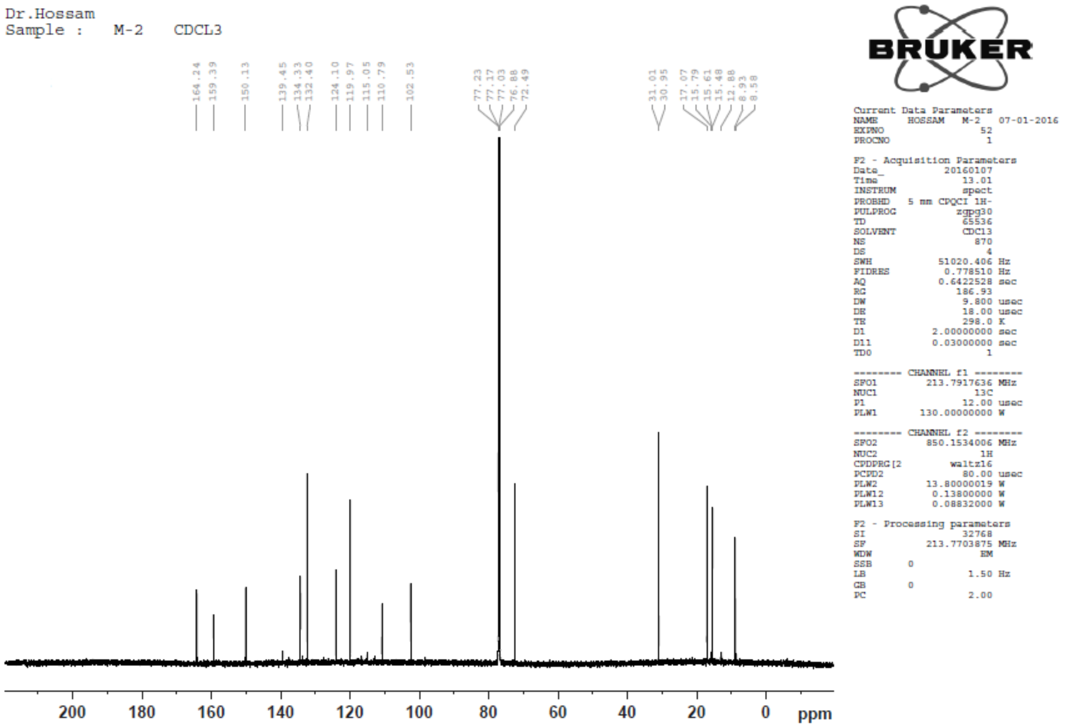

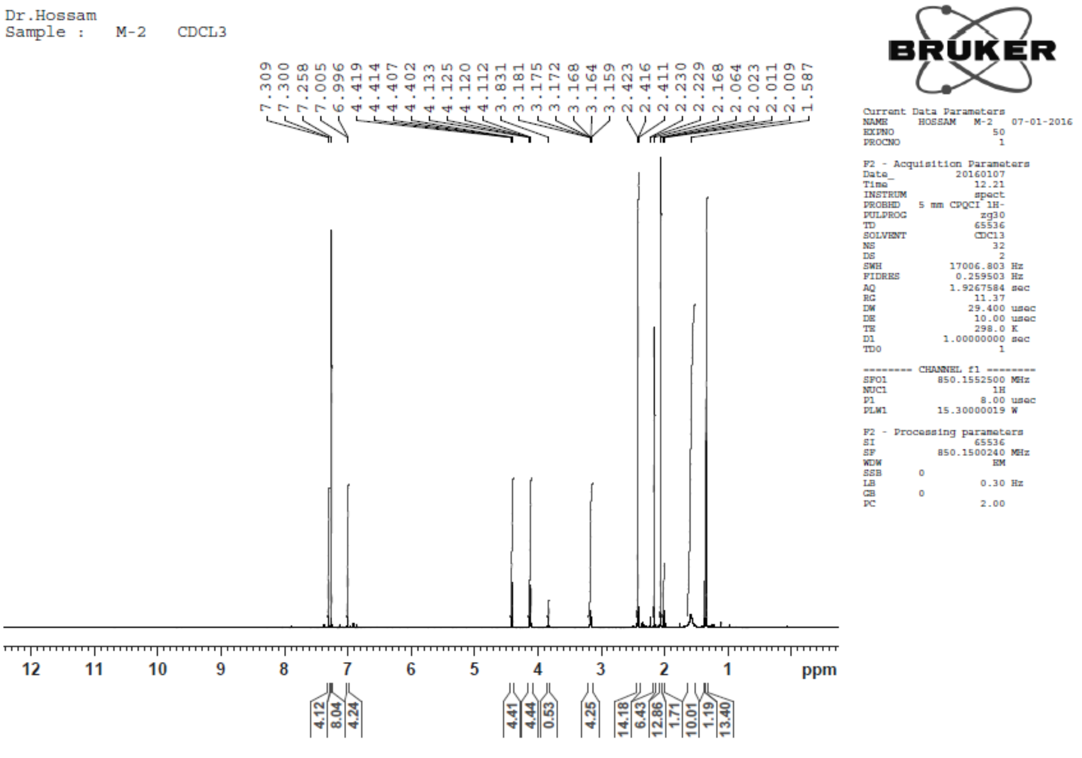

Supp. Fig 7. ^{1}H and [13] C NMR charts of compound 3

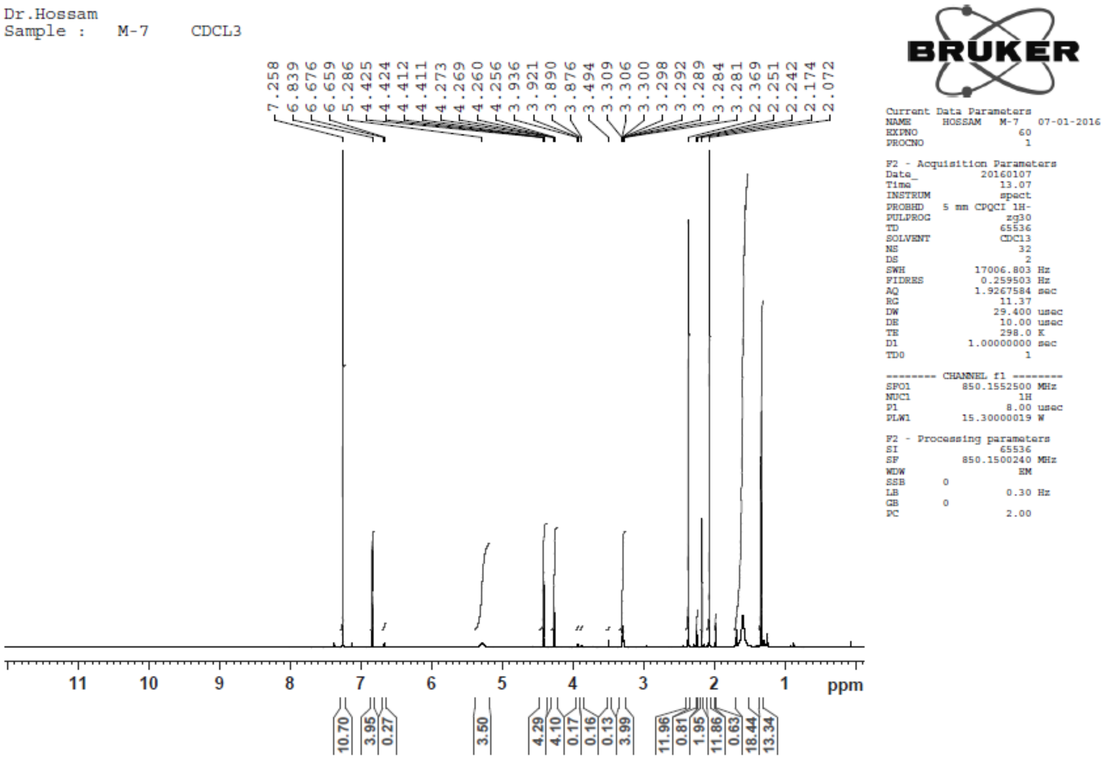

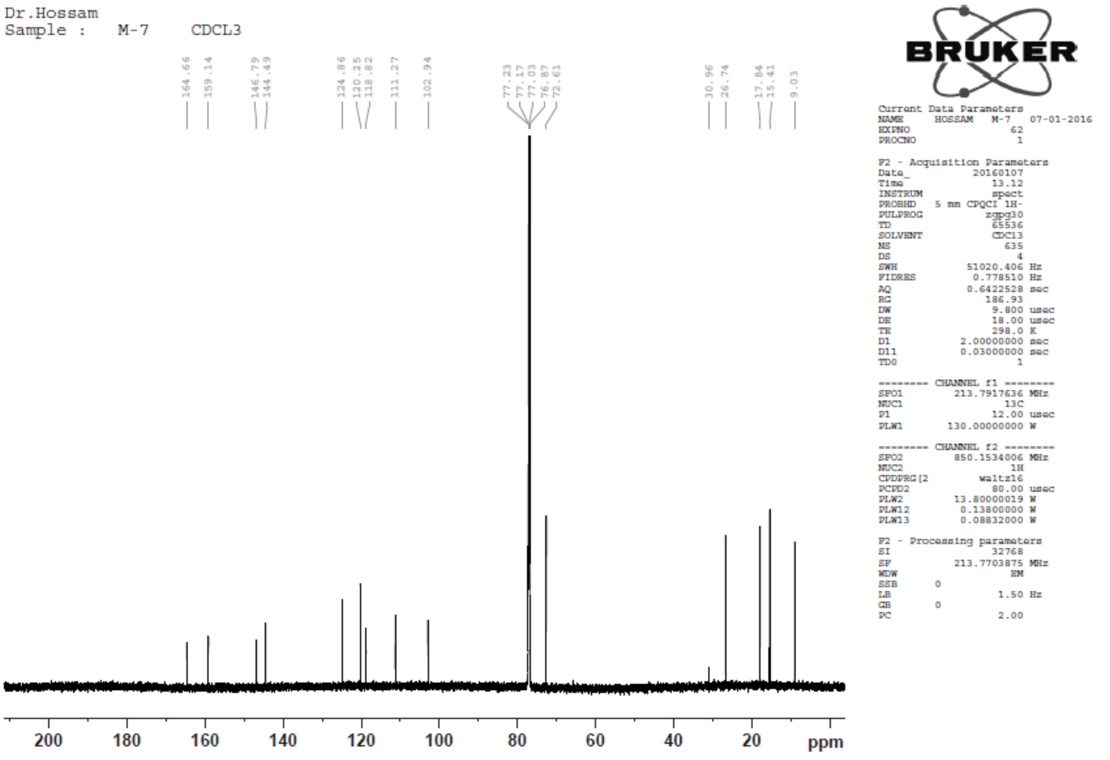

Supp. Fig 8. ^{1}H and ^{13}C NMR charts of compound 4

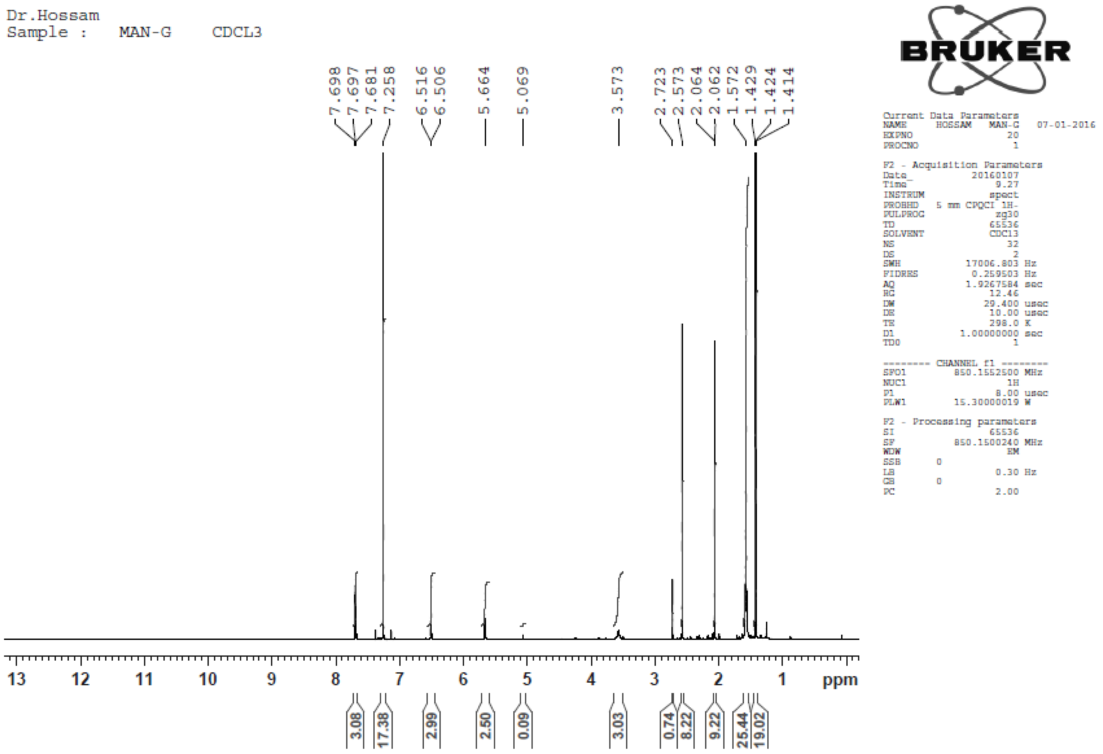

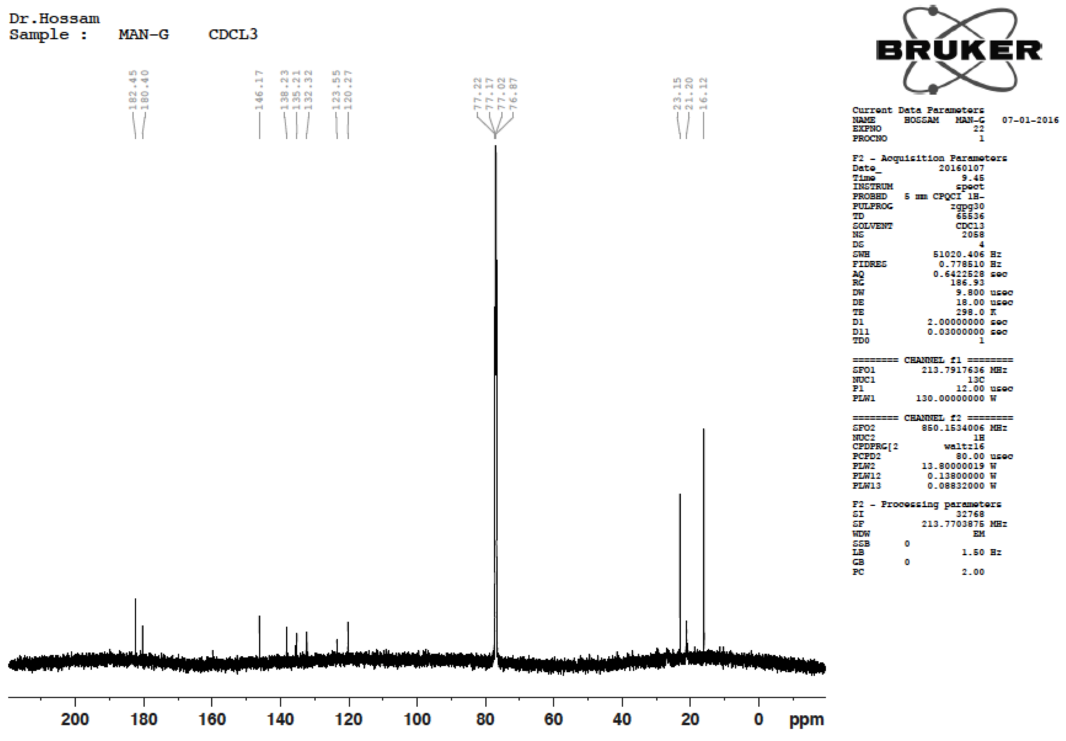

Supp. Fig 9. ^{1}H and ^{13} C NMR charts of compound 5

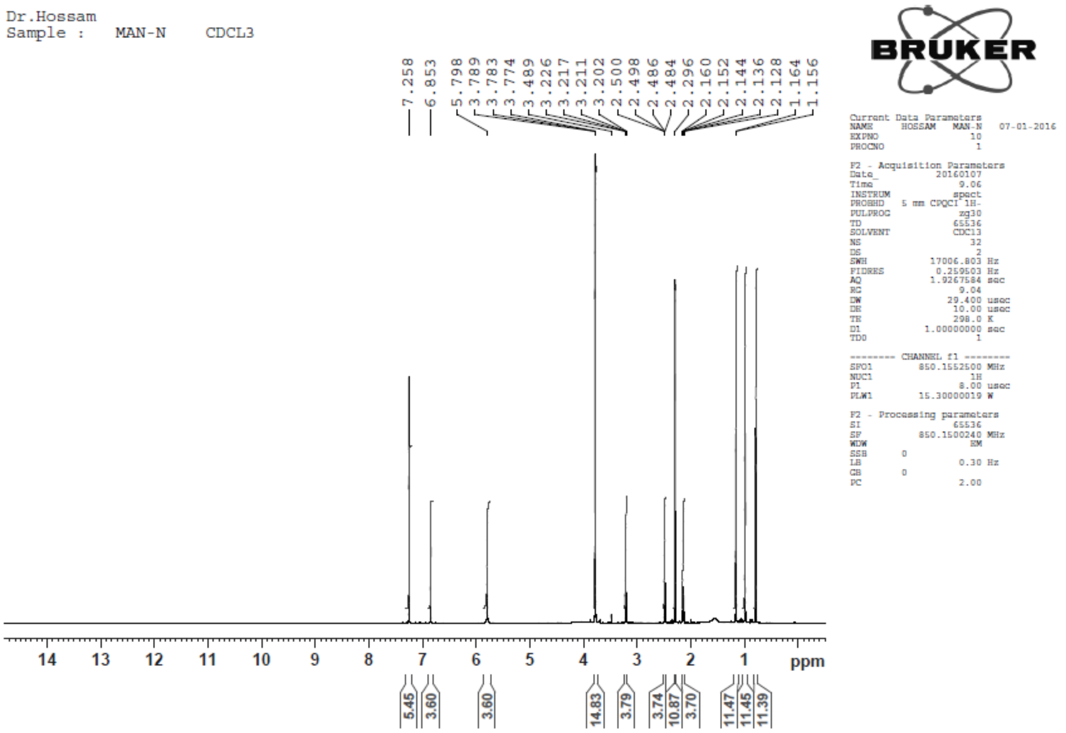

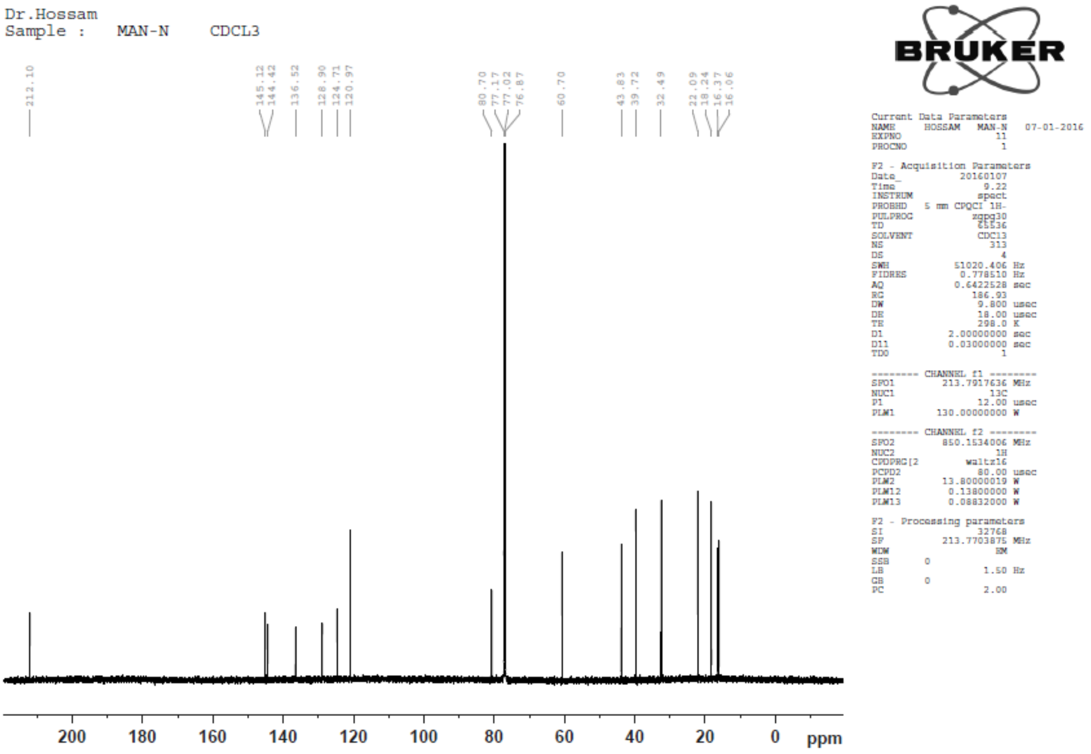

Supp. Fig 10. ^{1}H and [13] C NMR charts of compound 6

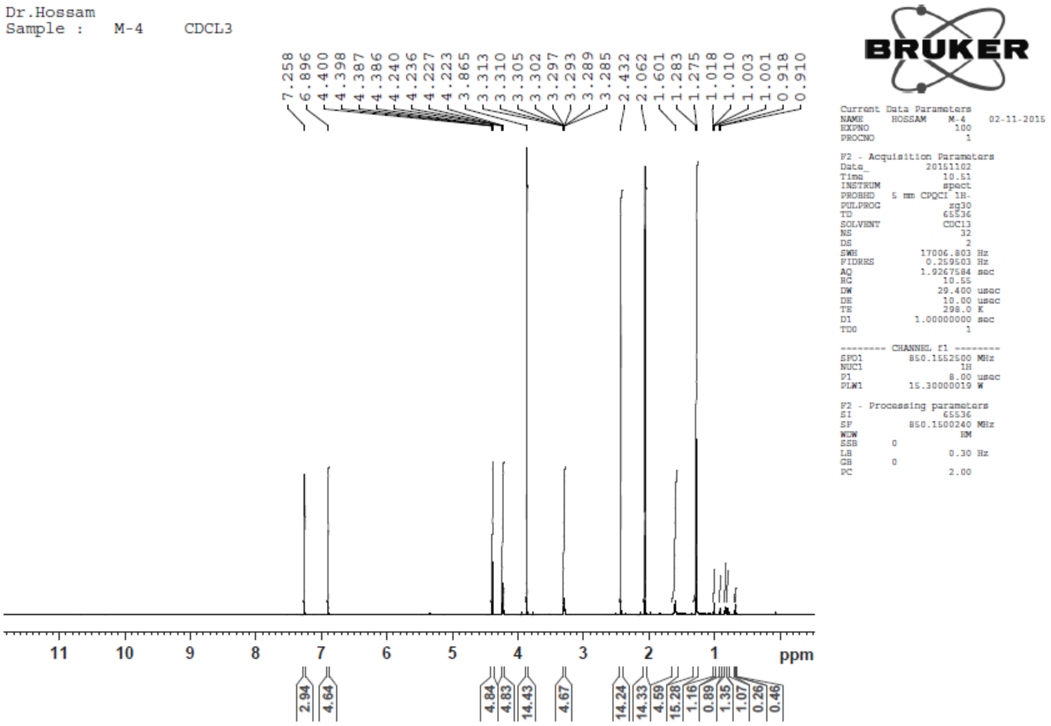

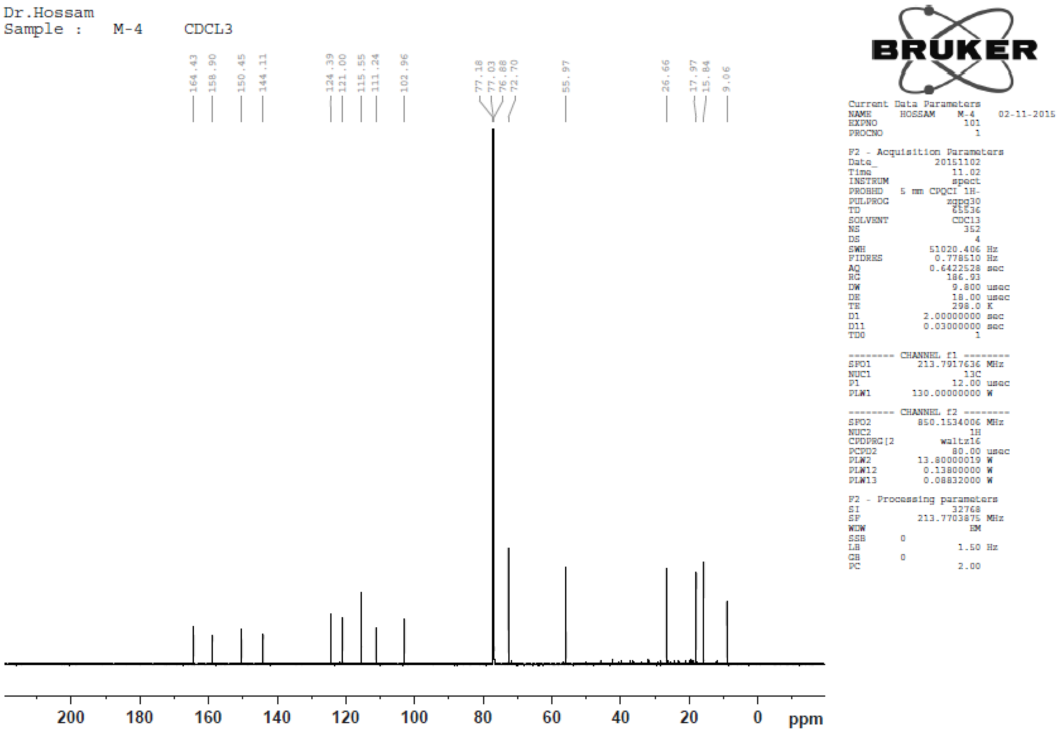

Supp. Fig 11. ^{1}H and [13] C NMR charts of compound 7

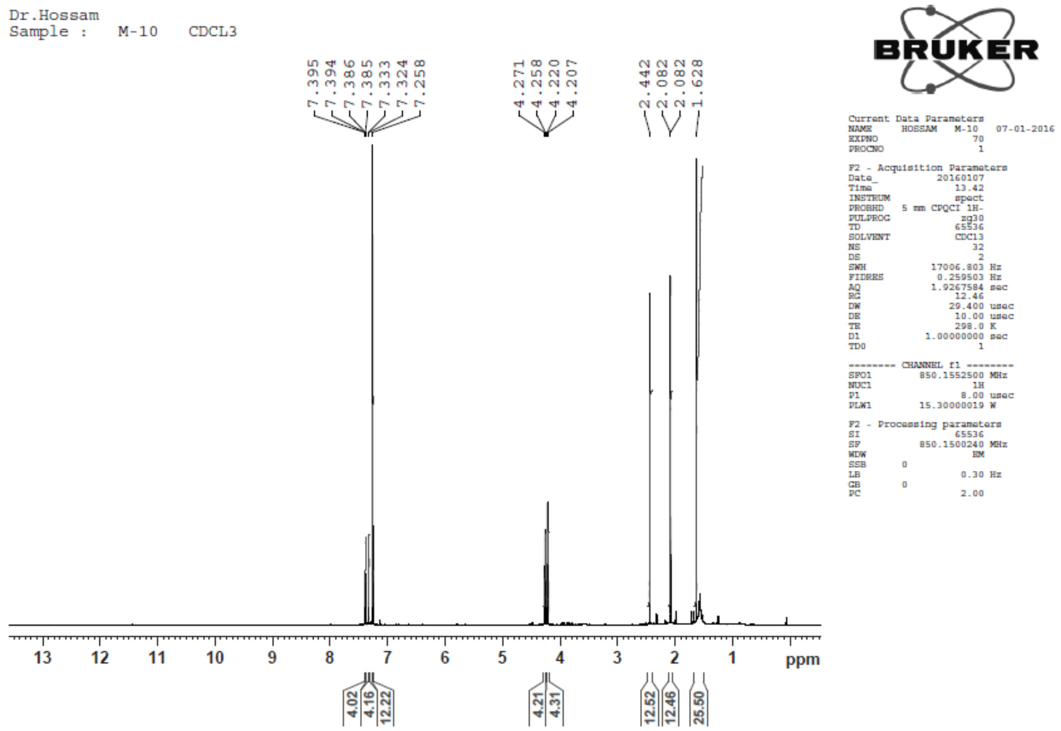

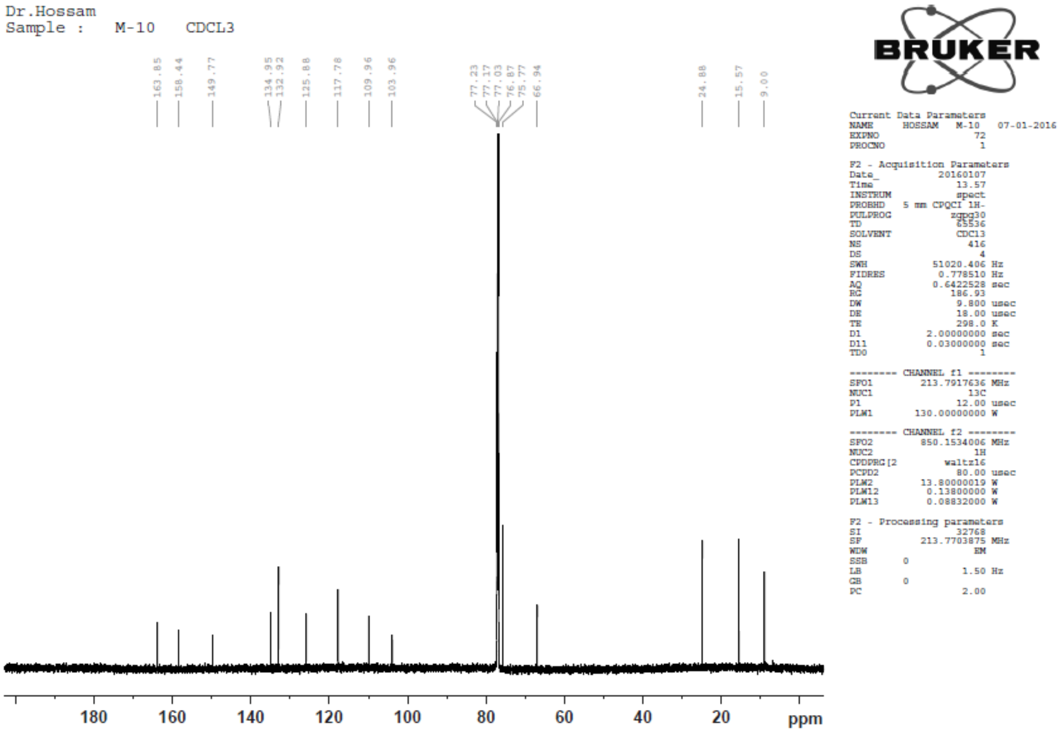

Supp. Fig 12. ^{1}H and ^{13} C NMR charts of compound 8
